# miR-29b-3p regulates cardiomyocytes pyroptosis in CVB3-induced myocarditis through targeting DNMT3A

**DOI:** 10.1186/s11658-024-00576-8

**Published:** 2024-04-20

**Authors:** Ya Wang, Zhengyang Zhang, Hui Li, Min Wang, Yuting Qiu, Lili Lu

**Affiliations:** Hubei Province Key Laboratory of Occupational Hazard Identification and Control, College of Medicine, Wuhan University of Science and Technology, Wuhan, 430065 Hubei People’s Republic of China

**Keywords:** Viral myocarditis, miR-29b-3p, DNMT3A, CVB3, Pyroptosis

## Abstract

**Background:**

Viral myocarditis (VMC) is a disease resulting from viral infection, which manifests as inflammation of myocardial cells. Until now, the treatment of VMC is still a great challenge for clinicians. Increasing studies indicate the participation of miR-29b-3p in various diseases. According to the transcriptome sequencing analysis, miR-29b-3p was markedly upregulated in the viral myocarditis model. The purpose of this study was to investigate the role of miR-29b-3p in the progression of VMC.

**Methods:**

We used CVB3 to induce primary cardiomyocytes and mice to establish a model of viral myocarditis. The purity of primary cardiomyocytes was identified by immunofluorescence. The cardiac function of mice was detected by Vevo770 imaging system. The area of inflammatory infiltration in heart tissue was shown by hematoxylin and eosin (H&E) staining. The expression of miR-29b-3p and DNMT3A was detected by quantitative real time polymerase chain reaction (qRT–PCR). The expression of a series of pyroptosis-related proteins was detected by western blot. The role of miR-29b-3p/DNMT3A in CVB3-induced pyroptosis of cardiomyocytes was studied in this research.

**Results:**

Our data showed that the expression of miR-29b-3p was upregulated in CVB3-induced cardiomyocytes and heart tissues in mice. To explore the function of miR-29b-3p in CVB3-induced VMC, we conducted in vivo experiments by knocking down the expression of miR-29b-3p using antagomir. We then assessed the effects on mice body weight, histopathology changes, myocardial function, and cell pyroptosis in heart tissues. Additionally, we performed gain/loss-of-function experiments in vitro to measure the levels of pyroptosis in primary cardiomyocytes. Through bioinformatic analysis, we identified DNA methyltransferases 3A (DNMT3A) as a potential target gene of miR-29b-3p. Furthermore, we found that the expression of DNMT3A can be modulated by miR-29b-3p during CVB3 infection.

**Conclusions:**

Our results demonstrate a correlation between the expression of DNMT3A and CVB3-induced pyroptosis in cardiomyocytes. These findings unveil a previously unidentified mechanism by which CVB3 induces cardiac injury through the regulation of miR-29b-3p/DNMT3A-mediated pyroptosis.

**Supplementary Information:**

The online version contains supplementary material available at 10.1186/s11658-024-00576-8.

## Introduction

Myocarditis is a kind of cardiovascular disease, which is considered to be the main cause of dilated cardiomyopathy [1] and sudden cardiac death [2]. It is crucial to note that VMC ranks as the third most common cause of related to cardiovascular death among young athletes (approximately 6%) [1]. Viral myocarditis (VMC), mainly triggered by cardiotropic virus infection, is the most prevalent type of myocarditis, leading to inflammatory damage to the myocardium [3–5]. Various viruses are associated with VMC, including enterovirus, adenovirus, human cytomegalovirus, human herpesvirus 6, parvovirus B19, hepatitis virus, and Epstein–Barr virus [6–8]. Among those pathogens, coxsackievirus B3 (CVB3) accounts for about 25% of viral myocarditis cases [2]. Research has indicated that in North America and Europe, patients with myocarditis/dilated cardiomyopathy often exhibit a higher prevalence of CVB3 infection [1]. Currently, there is no established regimen specifically designed for acute VMC other than conventional heart failure treatment and a period of physical rest. Thus, identifying the mechanisms of CVB3 induced myocardial injury will offer a novel perspective for the potential development of therapeutic strategies.

Different from apoptosis, pyroptosis was first discovered in macrophages [9, 10] and played a vital role in host defense against viral infection [11]. However, it has been documented that this process plays a role in the progression of various cardiovascular diseases (CVDs) [9, 12]. In many cases, inhibiting this process through drug or gene intervention has shown cardio-protective effects [12–14]. Therefore, the research and development of many therapeutic methods are based on pyroptosis regulation. Pyroptosis, a process instigated by the canonical caspase-1-dependent and noncanonical caspase-4/5/11-mediated pathways, of which the canonical pathway is typically activated by pathogen-associated molecular patterns (PAMPs) and danger-associated molecular patterns (DAMPs). In viral infection, PAMPs initiate the activation of the NLRP3 inflammasome, which is composed of NLRP3, ASC, and pro-Caspase-1. Then the NLRP3 inflammasome recruits pro-caspase-1 [12] and promotes it auto-cleavage into the activated form. This, in turn, results in the cleavage of interleukin-1β (IL-1β), interleukin-18 (IL-18), and gasdermin D (GSDMD), giving rise to an N-terminal fragment (GSDMD-N), which assembles into membrane pores, finally culminating in pyroptosis [15]. NLRP3 inflammasome-mediated pyroptosis assumes a significant role in the pathogenesis of VMC, and interventions targeting it have exhibited potential efficacy as therapeutic strategies [16, 17].

microRNAs (miRNAs) are a group of small, endogenous, noncoding RNA molecules that are highly conserved and primarily function to negatively regulate gene expression posttranscriptionally [18]. Numerous studies have emphasized the crucial role of miRNAs in various biological and pathological processes, including VMC. Experimental investigations have discovered that the expression of miR-155, miR-217, miR-543, and miR-21-5p is elevated in VMC mice model and correlated with the adverse outcome. Inhibition of those miRNAs can reduce myocardial cells injury in mice [19–21].

In this study, through transcriptome sequencing, we first identified the induction of miR-29b-3p in CVB3 induced VMC. Then the gain/loss-of-function experiments provided compelling evidence of miR-29b-3p’s mediation of the initiation and progression of VMC, primarily through the pyroptosis pathway. Moreover, DNMT3A has been verified as the specific gene targeted by miR-29b-3p during this process. Thus, the regulatory role of miR-29b-3p in CVB3-induced myocarditis was unveiled, which might offer novel insights into cardioprotective therapeutic targets for VMC treatment.

## Materials and methods

### Animal models

BALB/c mice (3–4 weeks old, male) [22, 23] were purchased from Wuhan Center for Disease Control and Prevention and were housed within the Laboratory Animal Center, Wuhan University of Science and Technology under a standard specific pathogen-free (SPF) environment. Body weight loss was used to assess the health of animals in this study. If mice lost up to 30% of their body weight before experiment ending, they will be euthanized and removed from the group to minimize unnecessary suffering. These experiments adhered to the provisions of the Ethical Committee of Wuhan University of Science and Technology (license no. 2023175), which conformed to the requirements of the Guide for the Care and Use of Laboratory Animals of the US National Institutes of Health (NIH Publication no.85–23, revised 1996) and the rules of the Basel Declaration.

To induce VMC, mice received either an intraperitoneal injection of CVB3 (10^4^ TCID50, 150 μL) or an equivalent volume of phosphate-buffered saline (PBS). Mice receiving antagomir treatment were injected on days 1, 3, and 5 following CVB3 infection. Mice were divided into the following groups: control, CVB3, CVB3 + antagomir negative control (n.c.), and CVB3 + antagomir.

### Cells and virus

We isolated primary cardiomyocytes from Sprague–Dawley (SD) rats aged 1–2 days old. Hearts were removed after euthanizing rats by decapitation, and the ventricles were finely minced and digested in Hank’s solution containing 0.05% trypsin, 0.05% type I collagenase, 0.05% type II collagenase and 1% bovine serum albumin 6–8 times for 5–10 min each time. Differential attachment and BrdU treated method were used to remove cardiac fibroblasts.

HL-1 and HeLa cells were grown in Dulbecco’s modified Eagle’s medium (DMEM) and supplemented with 10% fetal bovine serum (FBS) and 1% 100 μg/mL penicillin–streptomycin (Biosharp, BL505A) at a temperature of 37 ℃ in a 5% CO_2_ atmosphere.

The CVB3 virus (Nancy strain) was generously provided by Prof. Kailang Wu (College of Life Sciences, Wuhan University). For virus passage and titration, HeLa cells were used. The virus titer in our study was 1 × 10^7^ TCID50/0.1 mL.

For CVB3 infection, primary cardiomyocytes or HL-1 cells were exposed with varying concentration of CVB3 in serum-free media for 2 h at 37 ℃. Subsequently, they were rinsed with PBS and maintained in DMEM with 10% FBS.

### Echocardiography

To evaluate the cardiac function of mice, we conducted echocardiography while they were under anesthesia (1.5–1.8% isoflurane mixed gas inhalation anesthesia), employing the Vevo770 imaging system (VisualSonics Inc., Toronto, Canada) equipped with a 30 MHz high-frequency scanning head.

### Hematoxylin and eosin (H&E) staining

Heart tissues were fixed in 10% formaldehyde for 24 h, followed by paraffin embedding and sectioning into 5 μm slices. These sections underwent H&E staining using a standard method.

### Cell transfection

Lipofectamine 8000 (Beyotime, C0533) was utilized for cardiomyocytes transfection in accordance with the manufacturer’s guidelines. The miR-29b-3p inhibitor and mimic, as well as their respective negative controls, DNMT3A siRNA (si-DNMT3A) and its overexpression plasmid (phage-DNMT3A), were synthesized by Tsingke (Beijing, China). The transfection amounts for both inhibitor and mimic were set at 20 μM, while the siRNA for DNMT3A and phage-DNMT3A were at 20 μM and 2.5 μg, respectively.

### Quantitative real-time polymerase chain reaction (qRT–PCR)

Following the manufacturer’s guidelines, Magzol reagent (Magen, Guangzhou) was employed for total RNA extraction. To measure gene expression, SYBR Premix Ex Taq™ (YEASEN, Shanghai) was employed. For internal control, GAPDH and U6 served as benchmarks. The calculation of miRNAs or mRNA’s relative repression levels was carried out using the 2^−ΔΔCt^ method. Detailed information regarding the primers applied in this research can be found in Table 1.

**Table 1:** Primer sequences used in this study

Name	Sequences
miR-29b-3p-RT	5′-GTCGTATCCAGTGCAGGGTCCGAGGTATTCGCACTGGATACGACAACACT-3′
miR-29b-3p-FP	5′-CGCGTAGCACCATTTGAAATC-3′
miR-29b-3p-RP	5′-AGTGCAGGGTCCGAGGTATT-3′
U6-RT	5′-GTCGTATGCAGAGCAGGGTCCGAGGTATTCGCACTGCATACGACAAAATATGG-3′
U6-FP	5′-AAGGATGACACGCAAATTC-3′
U6-RP	5′-GAGCAGGGTCCGAGGT-3′
IL-6-FP	5′-TAGTCCTTCCTACCCCAATTTCC-3′
IL-6-RP	5′-TTGGTCCTTAGCCACTCCTTC-3′
IL-18-FP	5′-GACTCTTGCGTCAACTTCAAGG-3′
IL-18-RP	5′-CAGGCTGTCTTTTGTCAACGA-3′
DNMT3A-FP	5′-GAGGGAACTGAGACCCCAC-3′
DNMT3A-RP	5′-CTGGAAGGTGAGTCTTGGCA-3′
VP1-FP	5′-TTGCATATGGCCCAGTGGAAG-3′
VP1-RP	5′-TGTGGATCCTTATTGCCTAGTAGTGGTAACTC-3′
GAPDH-FP	5′-AGGTCGGTGTGAACGGATTTG-3′
GAPDH-RP	5′-TGTAGACCATGTAGTTGAGGTCA-3′
miR-29b-3p inhibitor	5′-AACACUGAUUUCAAAUGGUGCUA-3′
miR-29b-3p mimic-sense	5′-UAGCACCAUUUGAAAUCAGUGUU-3′
miR-29b-3p mimic-antisense	5′-CACUGAUUUCAAAUGGUGCUAUU-3′
miR-29b-3p-antagomir	5′- (mA)*(mA)*(mC)(mA)(mC)(mU)(mG)(mA)(mU) (mU)(mU) (mC)(mA) (mA)(mA) (mU)(mG) (mG)(mU) * (mG) * (mC) * (mU) * (mA)-3′
si-DNMT3A-sence	5′-CCAGAACUGUAAGAACUGCUU-3′
si-DNMT3A-antisence	5′-AAGCAGUUCUUACAGUUCUGG-3′

### Western blot

The extraction of total protein from cells or heart tissues utilizing RIPA lysate containing 1% PMSF (Biosharp, BL612A). We used the following primary antibodies: rabbit anti-ASC antibody (A1170), rabbit anti-IL-1β antibody (A1112), rabbit anti-NLRP3 antibody (A5652), rabbit anti-GSDMD/GSDMD-N antibody (A20197), rabbit anti-pro-Caspase-1/Caspase-1 antibody (A0964), and mouse anti-GAPDH antibody (AC002). All above primary antibodies sourced from Abclonal technology and the rabbit anti-DNMT3A primary antibody was purchased from Proteintech (20954-1-AP). The Pierce™ ECL western blotting substrate (Biosharp, BL520A) was used to visualize protein signals. The protein band gray density was quantified using ImageJ software (version 1.6065).

### Cell viability assay

Cell Counting Kit-8 (CCK-8) kit (Biosharp, BS350B) was used to evaluate cell viability.

HL-1 cells were seeded in a 96-well plate (0.5 × 10^4^ cells per well) and then treated with different doses of CVB3 (10, 100, 1000 TCID50) for 24, 48, and 72 h. After CVB3 treatment, the medium in each well was replaced with 100 μL CCK8 solution (containing 10 μL CCK8 regent and 90 μL DMEM basic medium) and incubated at 37 ℃ for 1 h. The absorbance at 450 nm (A_450_) of each well was measured, and the cell viability rate was calculated as described below. Cell viability (%) = (A_450_ of CVB3-treated group − A_450_ of blank)/(A_450_ of control group − A_450_ of blank) × 100%.

### Immunofluorescence

After the primary cardiomyocytes were isolated, cells were first fixed with 4% paraformaldehyde for a duration of 10 min, permeabilized using 0.5% Triton-X-100 for 10 min, and then subjected to blocking with 5% bovine serum albumin (BSA) for an hour. Following this, incubation was carried out overnight at 4 °C with primary antibodies. The primary antibody, anti-cTnT (1:100, Bioss, BS10648R), was used in this study.

### Grade of myocarditis

The severity of myocarditis was evaluated according to H&E section of heart tissue and scored from 0 to 4 points as previously described [24]. 0, no inflammation; 1, one to five obvious mononuclear inflammatory lesions and with an inflammatory infiltration area of no more than 5% of total cross-sectional area; 2, more than five different lesions and with an inflammatory infiltration area between 5% and 20%; 3, diffuse lesions involving an area of more than 20%; 4, diffuse lesions accompanied with necrosis. Lesions were characterized as regions of inflammation and/or cardiomyocyte necrosis and loss.

### Statistical analysis

Data were expressed as mean ± standard deviation (mean ± SD). Unpaired *t*-test and one-way ANOVA test was applied for two groups and multiple groups comparison respectively. GraphPad Prism 7.0 was utilized for all analyses. *P* < 0.05 was considered statistical significance.

## Results

### CVB3 could increase pyroptosis in myocarditis in vivo and in vitro

**Fig. 1:**
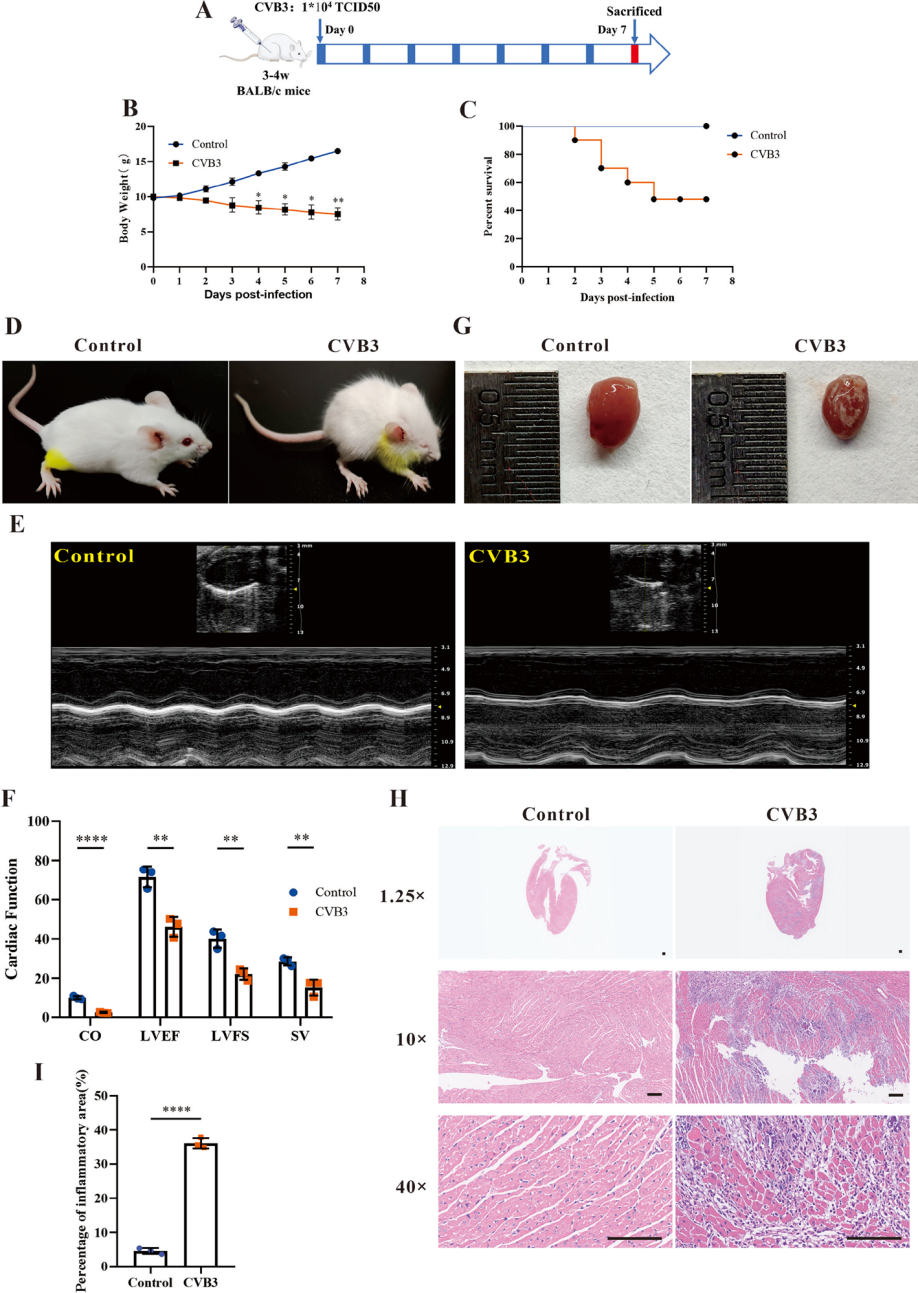
CVB3 induced VMC in mice. The male BALB/c mice were treated with 1 × 10^4^ TCID50 CVB3 and then sacrificed on day 7 p.i. **A** A schematic diagram of the viral myocarditis modeling process induced by CVB3. **B** The body weight change of mice after CVB3 infection. **C** The survival rate of mice following CVB3 infection. **D** The appearance of mice on day 7 p.i. **E** M-mode images of heart and **F** The cardiac function was evaluated using echocardiography. **G** Heart appearance of mice on day 7 post CVB3 infection. **H** H&E staining of the heart tissue sections; the magnifications were 1.25 × , 10 × , 40 × , respectively, and the scale bar was 100 μm. **I** Quantification of inflammatory areas based on the images shown in **H**. *N* = 3, *p < 0.05, **p < 0.01, and ****p < 0.0001

In this research, we first established the VMC mice model induced by CVB3 (Fig. 1A). From day 4 postinfection (p.i.), a notable body weight reduction was observed in CVB3-infected mice (Fig. 1B). Accompany with the body weight loss, the mortality increased. On day 5 p.i., the survival rate of CVB3 mice decreased to 50% (Fig. 1C). The CVB3 group mice were lethargic with activity decreasing and hair luster loss (Fig. 1D). The results of echocardiography indicated that the cardiac function of the CVB3 group was impaired (Fig. 1E, F). Subsequently, the hearts were isolated after euthanizing mice by decapitation on day 7 p.i. The heart appearance of CVB3 group manifested multiple white plaques, which were snowflake-like (Fig. 1G). H&E staining results indicated the inflammatory infiltration exist there. According to the extent of inflammatory infiltration in heart tissues, the myocarditis grading of CVB3 group was four points (Fig. 1H, I). These findings indicated that the establishment of the in vivo VMC model was successful.

**Fig. 2:**
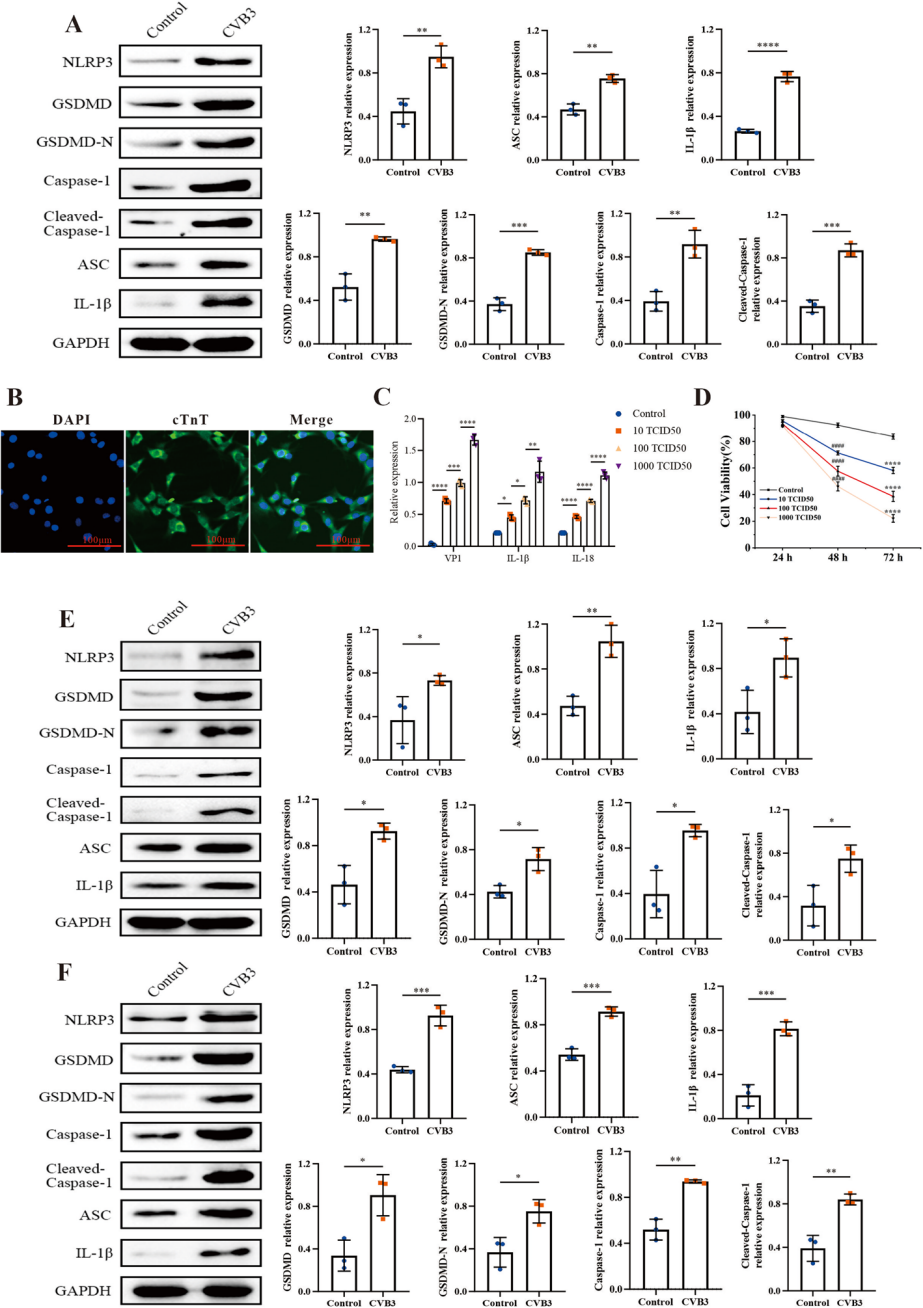
Pyroptosis increased in both in vivo and in vitro VMC models induced by CVB3. **A** The expressions of pyroptosis related proteins in heart tissue. **B** Primary cardiomyocytes isolated from SD rats were identified by immunofluorescence staining. The primary antibody of anti-cTnT was applied. The scale bar was 100 μm. **C** The total RNA was extracted from the primary cardiomyocytes exposed to varying concentrations of CVB3 and the expressions of inflammatory cytokines IL-1β and IL-18 were evaluated by qRT–PCR. **D** The cell viability was detected by CCK8. ####*p* < 0.0001 represents the comparison with control at 48 h; ****p < 0.0001 represents the comparison with control at 72 h. **E**, **F** The expressions of pyroptosis-related proteins in CVB3 treated primary cardiomyocytes and HL-1 cells were measured using western blotting. *N* = 3, *p < 0.05, **p < 0.01, ***p < 0.001, and ****p < 0.0001

Pyroptosis is an inflammatory form of programmed cell death (PCD), which involved in the pathogenesis of VMC [3, 25]. The pyroptosis levels of CVB3 infected were assessed in vivo and in vitro. Firstly, the expressions of pyroptosis-associated proteins in heart tissue were evaluated by western blot. The expressions of NLRP3, ASC, cleaved caspase-1, GSDMD-N, and IL-1β were upregulated in CVB3-infected mice (Fig. 2A). That indicated cardiomyocytes pyroptosis could be induced by CVB3 infection in VMC mice model. The levels of pyroptosis in CVB3-infected cells were evaluated in this study using the HL-1 cell line and primary cardiomyocytes. The primary cardiomyocytes were isolated from SD rats, as described in the Methods, and their purity was confirmed to be 98% through immunofluorescence staining using anti-cTnT (Fig. 2B). In this study, the levels of IL-1β and IL-18, two crucial cytokines of pyroptosis, were measured under varying concentrations virus treatment. The qRT–PCR results showed that as the virus copies increased, the levels of IL-1β and IL-18 mRNA also increased accordingly (Fig. 2C). To further demonstrate that CVB3 can induce pyroptosis, a form of inflammatory cell death, the viability of cardiomyocytes was measured using a CCK8 assay after treatment with varying doses of the virus for different time durations. As shown in Fig. 2D, CVB3 infection lead to a sharp decrease in cell viability. These results were confirmed by western blot as well (Fig. 2E, F). That suggested CVB3 infection can induce pyroptosis in vitro.

### MiR-29b-3p was involved in CVB3 induced cardiomyocytes pyroptosis

**Fig. 3:**
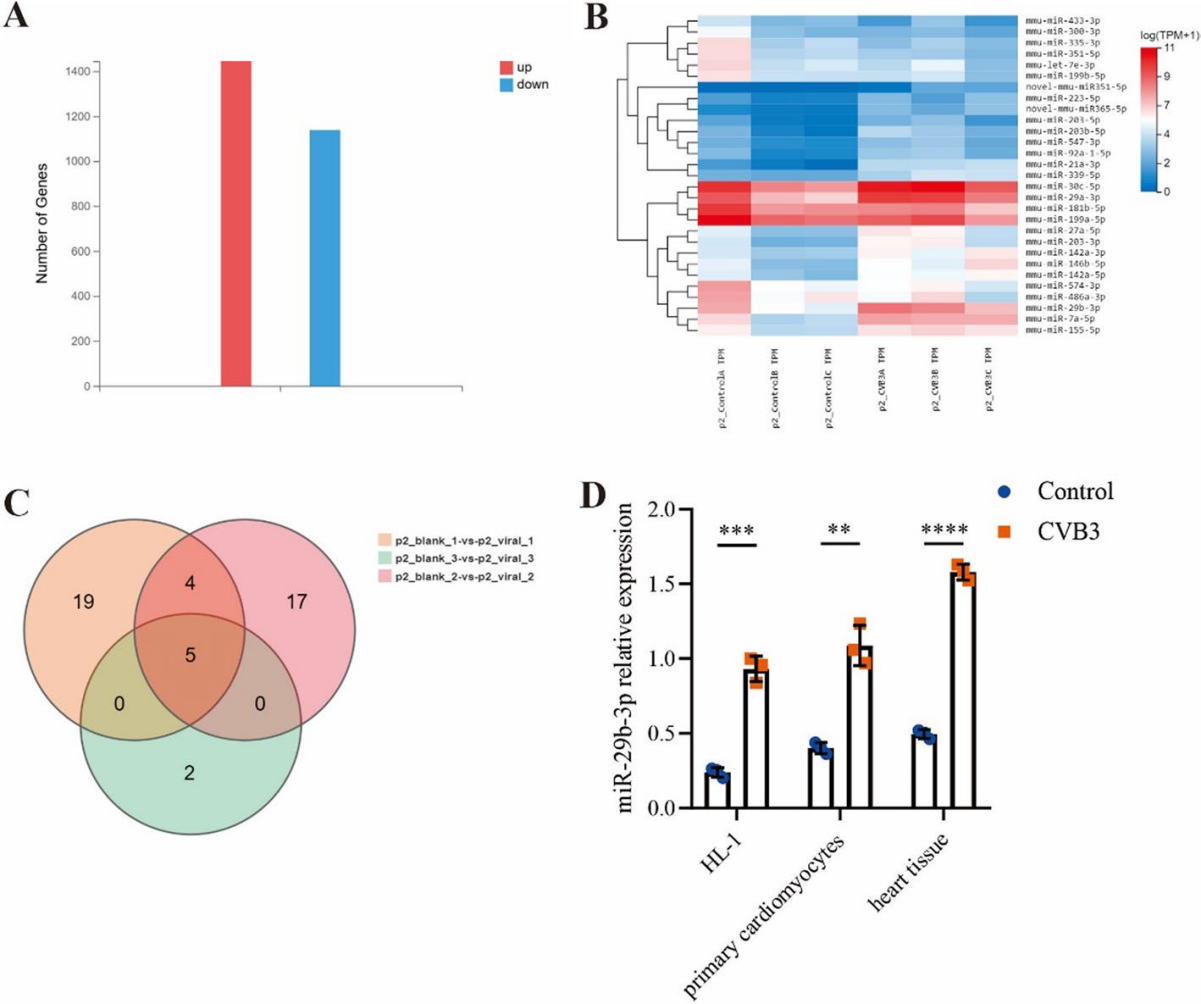
MiR-29b-3p was significantly upregulated in VMC models. The total RNA, extracted from VMC mice and the control, was applied to transcriptome sequencing (*N* = 3). **A** Compared with the control group, there were 1444 miRNAs increased and 1138 miRNAs decreased in VMC mice heart tissues. **B** The most differentially expressed miRNAs were displayed through clustering heat map. **C** The Venn diagram indicated that five miRNAs have consistent expression trends in all samples.** D** The expression levels of miR-29b-3p in HL-1 cells, primary cardiomyocytes, and heart tissue were evaluated by qRT–PCR. *N* = 3, **p < 0.01, ***p < 0.001, and ****p < 0.0001

MiRNAs involve with many pathological processes in diseases including VMC [26–28]. To elucidate the potential miRNA mechanisms in the pathogenesis of VMC, we conducted transcriptome sequencing (Beijing Genomics institution, BGI) to compare the expression difference of miRNAs within heart tissues between VMC mice and the control group. The results of differential analysis illustrated that there were 1444 miRNAs increased and 1138 miRNAs decreased in VMC mice heart tissues when compared with the control. Based on |log_2_fold change (FC)|  > 1 and p < 0.05, five miRNAs (miR-203-3p, miR-203b-5p, miR-21a-3p, miR-29b-3p, and miR-7a-5p) were identified as having the most significant differences through clustering heat map and Wayne map analysis (Fig. 3A–C). This analysis identified miR-29b-3p as one of the most significantly altered miRNAs. Subsequently, we validated miR-29b-3p’s expression levels both in vivo and in vitro using qRT–PCR, as illustrated in Fig. 3D. Previous studies proved miR-29b-3p could regulate the inflammatory response and be associated with myocardial injury and fibrosis in myocardial infarction [29–33]. However, the role of miR-29b-3p in VMC remains unclear.

**Fig. 4:**
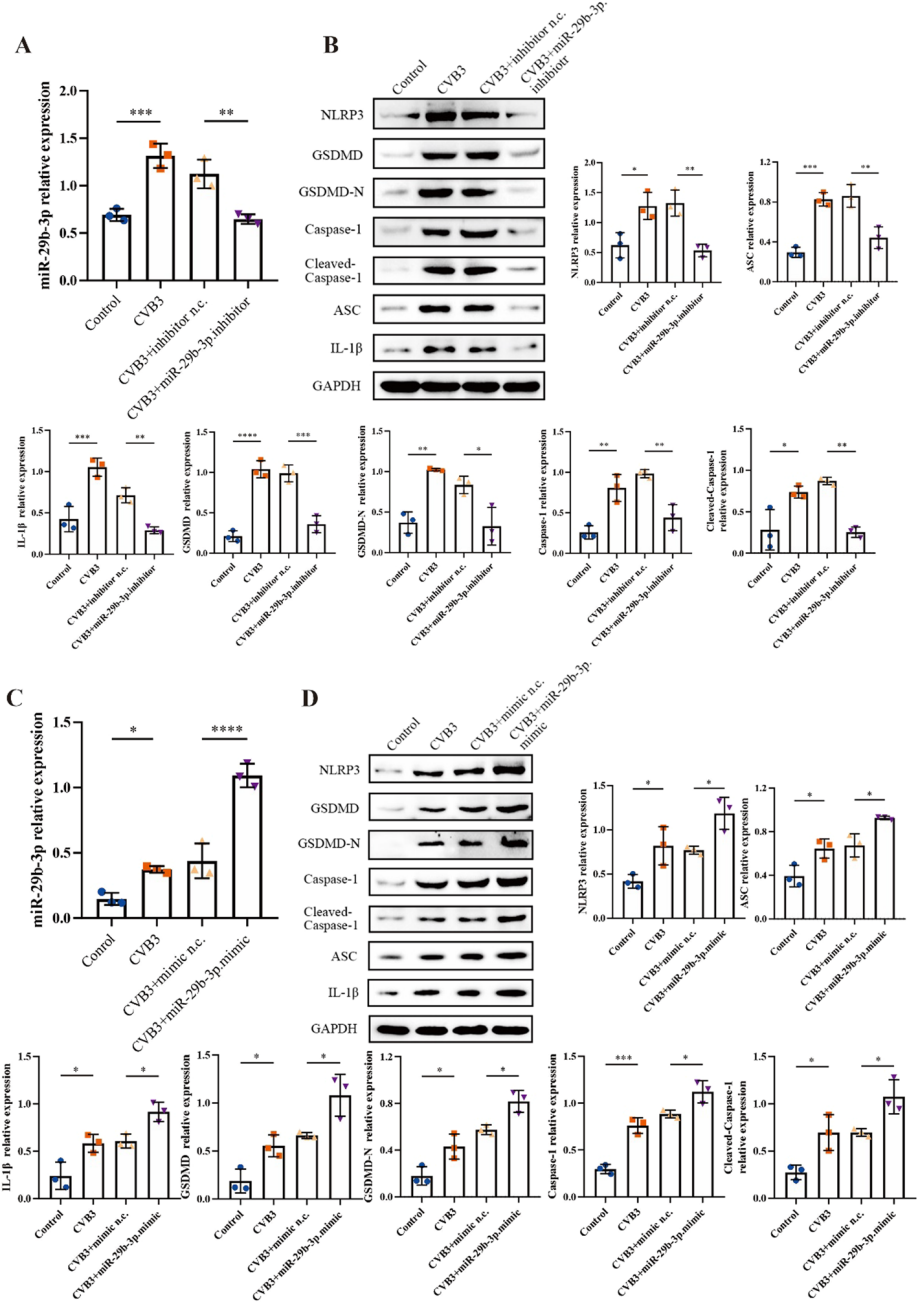
The role of miR-29b-3p in CVB3-induced cardiomyocyte pyroptosis. Primary cardiomyocytes were transfected with 20 μM miR-29b-3p inhibitor/mimic or the negative control (inhibitor/mimic n.c.), respectively, after CVB3 treatment. **A**, **C**The relative expression of miR-29b-3p was measured by qRT–PCR. U6 served as the internal reference. **B**, **D** The expressions of pyroptosis associated proteins were assessed by western blotting. *N* = 3, *p < 0.05, **p < 0.01, ***p < 0.001, and ****p < 0.0001

To clarify the role of miR-29b-3p in the progression of VMC, the miR-29b-3p inhibitor and mimic were synthesized by Beijing Tsingke Biotech. The inhibitor and mimic could effectively regulate the expression of miR-29b-3p within cells, as demonstrated in Fig. 4A and C. Using these miRNA regulation tools, we modulated miR-29b-3p expression in primary cardiomyocytes, then assessed the pyroptosis related proteins levels. The western blot results illustrated that the extent of pyroptosis caused by CVB3 infection was alleviated by miR-29b-3p inhibitor and was aggravated by miR-29b-3p mimic (Fig. 4B and D). That means miR-29b-3p might be a key factor in the pyroptosis of cardiomyocytes triggered by CVB3.

### Downregulation of miR-29b-3p ameliorated CVB3 induced myocarditis and pyroptosis in mice

**Fig. 5:**
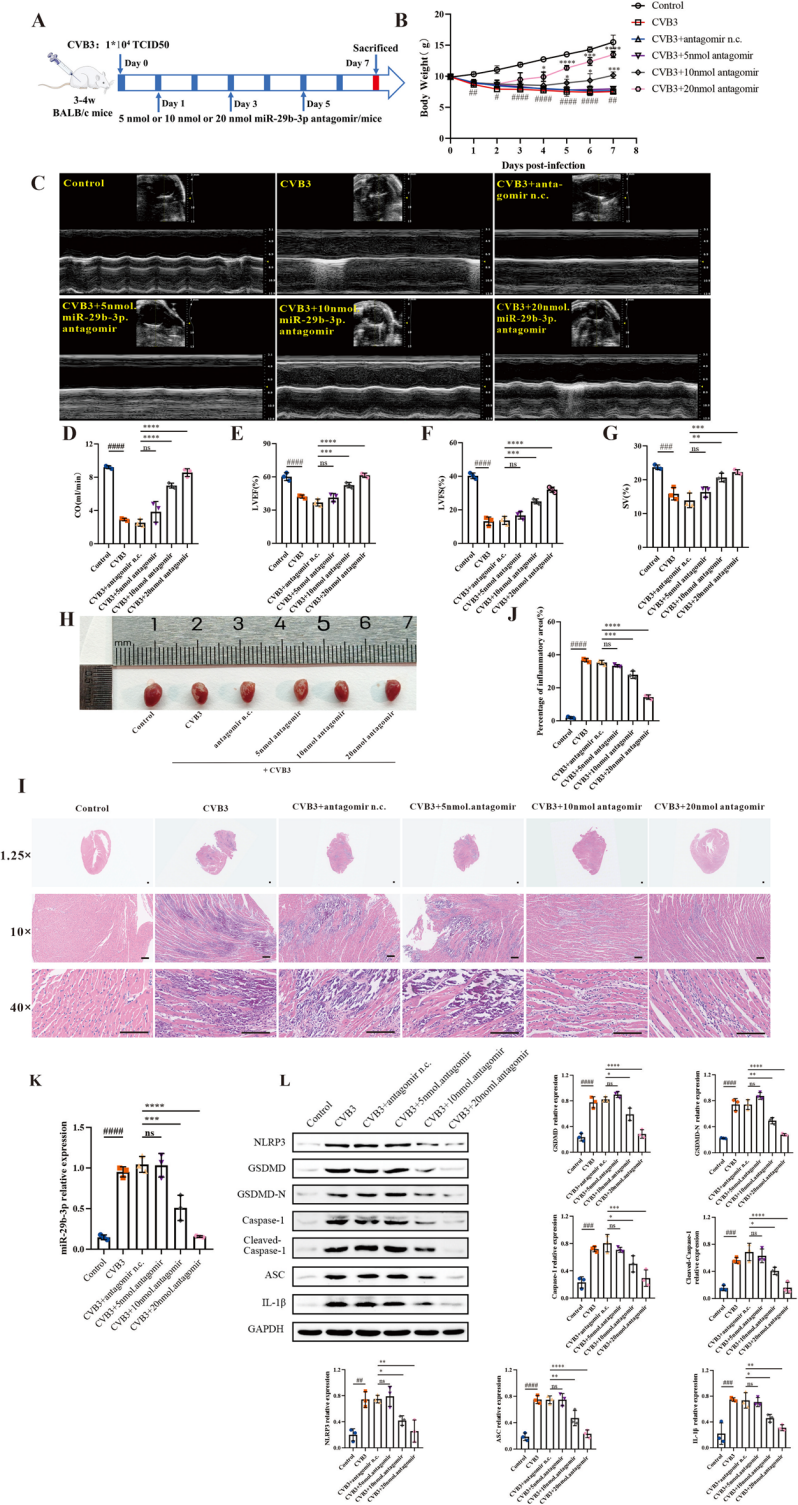
Downregulation of miR-29b-3p-ameliorated, CVB3-induced VMC through decreasing pyroptosis in vivo. **A** A schematic diagram of miR-29b-3p antagomir treatment in VMC model induced by CVB3. The mice were inoculated with CVB3 on day 0 and then treated with indicated concentrations of miR-29b-3p antagomir (5, 10, 20 nM) on days 1, 3, and 5, respectively. **B** The body weight change of VMC mice after miR-29b-3p antagomir treatment. **C** M-mode images of heart and **D**–**G** the parameters of cardiac function were evaluated using echocardiography. **H** Heart appearance of VMC mice treated with miR-29b-3p antagomir on day 7. **I** H&E staining of the heart tissue sections; the magnifications were 1.25 × , 10 × , and 40 × , respectively, and the scale bar was 100 μm. **J** The inflammatory lesions were quantified using Image-Pro Plus 6.0 software. **K** Total RNA was extracted from heart tissue and the expression of miR-29b-3p was evaluated by qRT–PCR. **L** Total proteins were extracted from heart tissue and the expressions of pyroptosis proteins were analyzed by western blotting. *N* = 3, #*p* < 0.05, ##*p* < 0.01, ###*p* < 0.001, and ####*p* < 0.0001; *ns* significance is annotated as not significant, *p < 0.05, **p < 0.01, ***p < 0.001, ****p < 0.0001

To elucidate the function of miR-29b-3p in VMC, the mice were treated with different doses of antagomiR-29b-3p (5, 10, 20 nM) by intraperitoneal injection on day 1, 3, 5 postinfection of CVB3 (Fig. 5A). The body weight records indicated the mice in the CVB3 and CVB3 + antagomir n.c. groups experienced a gradual decrease in body weight after viral treatment. While the mice treated with 10 or 20 nM antagomiR-29b-3p started to regain body weight from day 4 or 5 p.i., this increase was statistically significant compared with the CVB3 + antagomir n.c. group on day 7 (Fig. 5B). The echocardiography results also proved that the cardiac function of the groups treated with antagomiR-29b-3p was markedly improved as the concentration of antagomiR-29b-3p increased (Fig. 5C). The left ventricular cardiac output (CO), left ventricular ejection fraction (LVEF), left ventricular fraction shortening (LVFS), and stroke volume (SV) were notably enhanced, particularly in the 20 nM miR-29b-3p antagomir group, when compared with the CVB3 and CVB3 + antagomir n.c. groups (Fig. 5D–G). Moreover, the appearance of heart tissues (Fig. 5H) and extent of inflammatory infiltration (Fig. 5I, J) correlated with the changes in cardiac function. These findings provide evidence that miR-29b-3p antagomir alleviated myocarditis.

Subsequently, we extracted total RNA and proteins from the heart tissue of mice treated with antagomiR-29b-3p, evaluated the expressions of miR-29b-3p and pyroptosis-related proteins. Figure 5K demonstrates that the antagomiR-29b-3p downregulated the expression of miR-29b-3p in a dose-responsive manner in vivo. At the same time, the level of pyroptosis in heart tissue decreased consistently with the increasing concentration of antagomiR-29b-3p (Fig. 5L). That means antagomiR-29b-3p improves myocarditis by reducing cardiomyocyte pyroptosis.

### DNMT3A is the target of miR-29b-3p in CVB3 induced pyroptosis

**Fig. 6:**
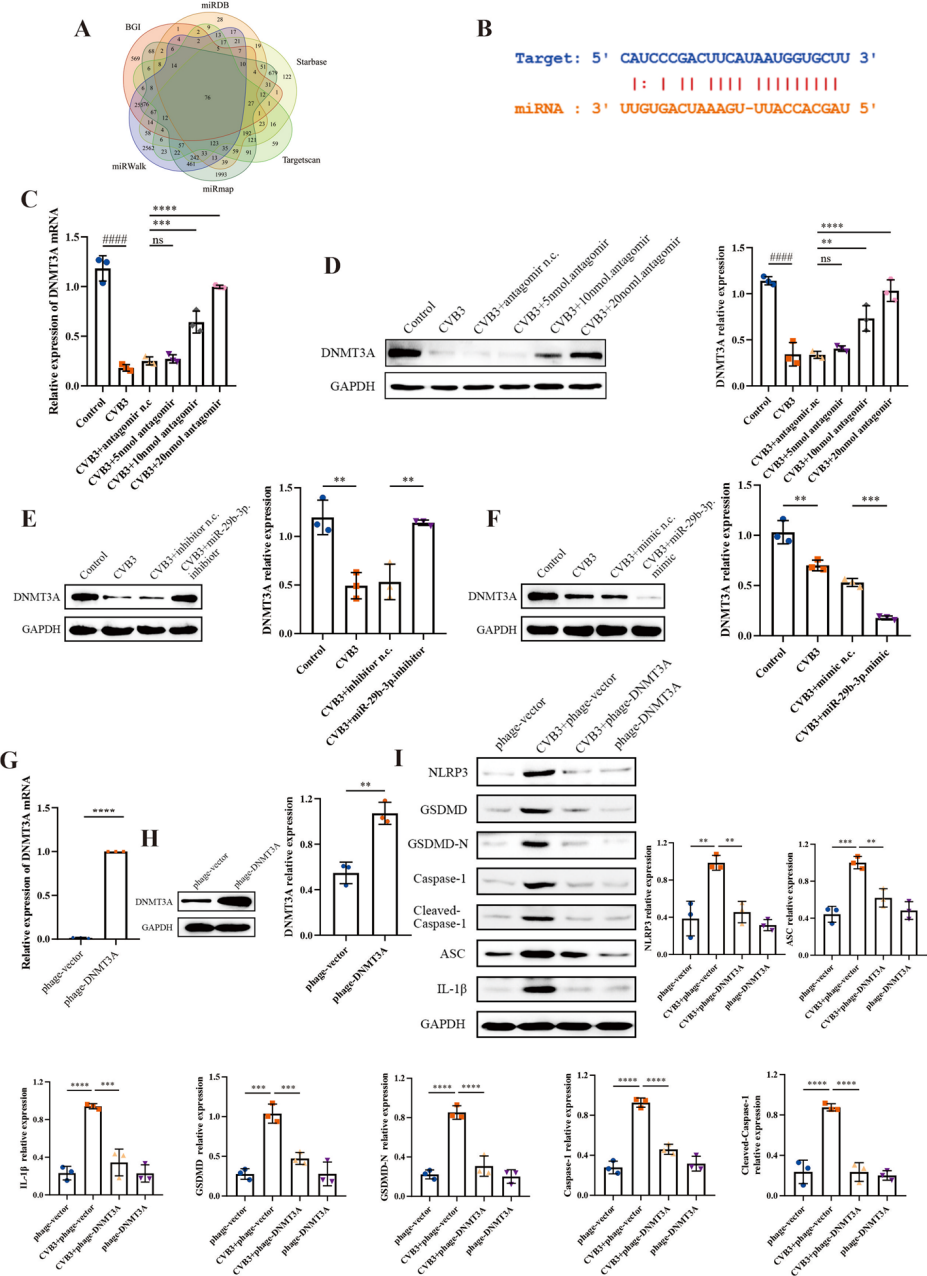
DNMT3A acted as a target gene of miR-29b-3p in CVB3-induced myocarditis and reduced pyroptosis.** A** Bioinformatic analysis predicted the potential targets of miR-29b-3p. **B** The seed region of miR-29b-3p matches the sequence (nucleotides 825–831) on 3′ UTR of DNMT3A. **C**, **D** Total RNA and protein were extracted from heart tissues to detect the expression levels of DNMT3A. **E**, **F** The primary cardiomyocytes were transiently transfected with 20 μM miR-29b-3p inhibitor or mimic. A total of 48 h later, total protein was extracted and subjected to western blotting. The relative expression of DNMT3A was represented as a histogram and normalized using GAPDH. **G**, **H** Primary cardiomyocytes were transfected with 2.5 μg plasmid (phage-DNMT3A or phage-vector) for 48 h, and then total RNA and protein were extracted to evaluate DNMT3A expression. **I** CVB3-infected cardiomyocytes were transfected with 2.5 μg plasmid as indicated. After 48 h, pyroptosis level was evaluated by western blotting. *N* = 3, ####*p* < 0.0001,** *p* < 0.01, ***p < 0.001, and ****p < 0.0001

To investigate the molecular mechanism of miR-29b-3p promoting the progression of VMC, several bioinformatics tools including TargetScan, Starbase, miRWalk, miRmap, BGI, and miRDB were utilized to identify the potential targets of miR-29b-3p. DNA methyltransferase 3A (DNMT3A) was considered as one of the candidates after bioinformatics analysis (Fig. 6A). The results revealed a matching sequence between miR-29b-3p and the 3′ UTR of DNMT3A (nucleotides 825–831), as depicted in Fig. 6B. Notably, several studies have established DNMT3A as a direct target of miR-29b-3p, as evidenced by dual-luciferase reporter assays [33–36]. To further validate DNMT3A as a target of miR-29b-3p in CVB3 induced pyroptosis, the in vivo and in vitro functional analysis was performed. The mRNA and protein expression levels of DNMT3A were examined in mice heart tissue. The results from qRT–PCR and western blot revealed a dose-dependent increase in DNMT3A expression with increasing levels of miR-29b-3p antagomir (Fig. 6C-D). Then, as shown in Fig. 6E and F, the expression of DNMT3A was also found to be regulated by miR-29b-3p inhibitor/mimic in CVB3 infected cardiomyocytes. The expression of DNMT3A dramatically decreased with the treatment of miR-29b-3p mimic. When the expression of miR-29b-3p was downregulated by the inhibitor, the expression of DNMT3A increased, which was consistent with the results in vivo. These findings provide compelling evidence that DNMT3A is directly targeted by miR-29b-3p in our study.

However, the function of DNMT3A in pyroptosis was still unknown. To investigate this, the overexpression plasmid (phage-DNMT3A) was designed to regulate the cellular expression of DNMT3A. qRT–PCR and western blot results displayed that phage-DNMT3A could effectively upregulate DNMT3A expression (Fig. 6G, H). Furthermore, it was observed that DNMT3A could reverse CVB3 induced pyroptosis in vitro (Fig. 6I). These results indicate that DNMT3A contributes to the damage of cardiomyocytes induced by CVB3 and acts as a protective factor.

### MiR-29b-3p regulated pyroptosis via DNMT3A in CVB3-induced myocarditis

**Fig. 7:**
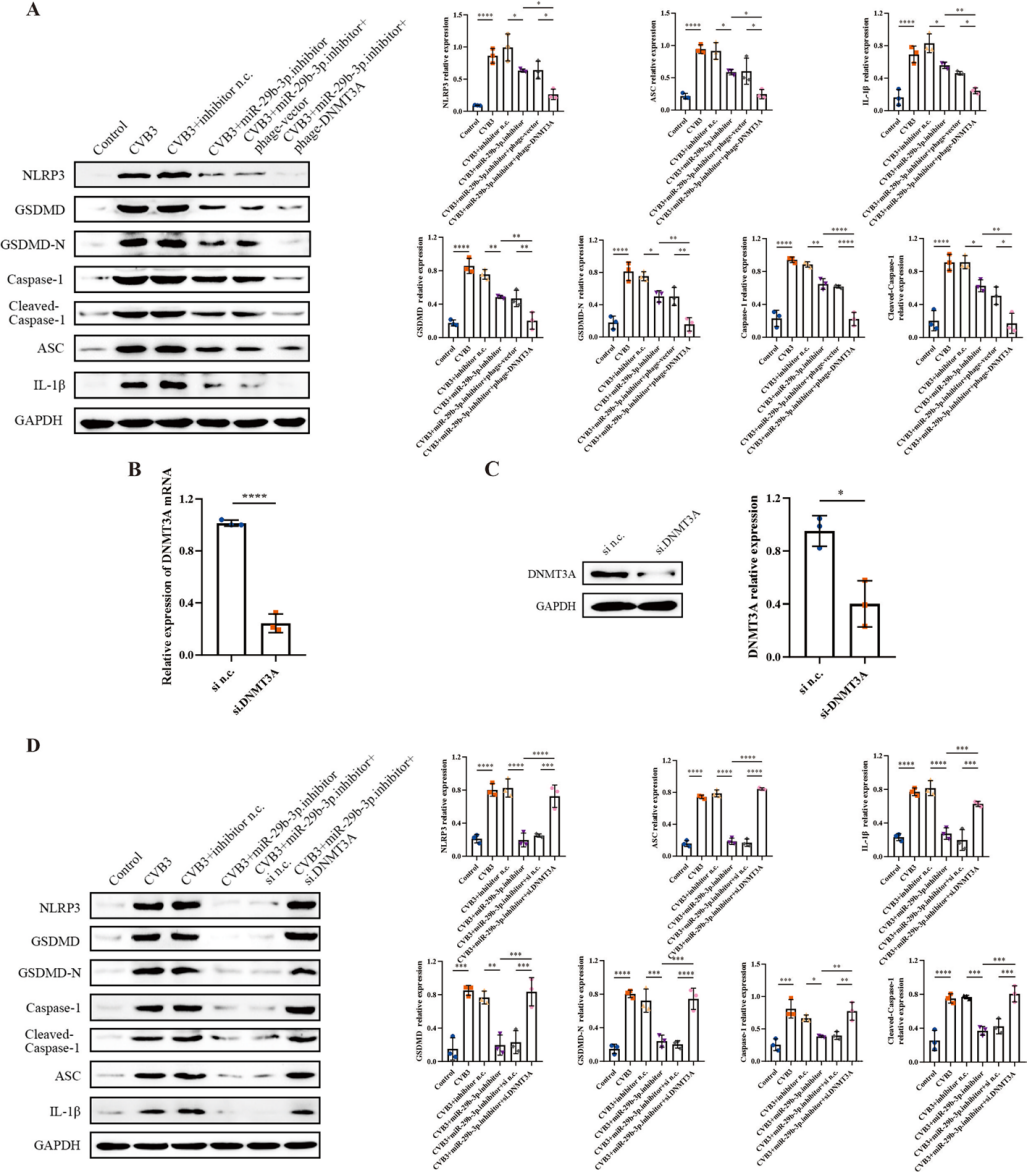
miR-29b-3p regulated cardiomyocyte pyroptosis via DNMT3A. The primary cardiomyocytes were exposed to CVB3 for 6 h, then co-transfected with phage-DNMT3A or phage-vector and miR-29b-3p inhibitor for another 48 h. **A** The expression levels of proteins related to pyroptosis. **B**, **C** Primary cardiomyocytes were transfected with 20 μM of si-DNMT3A or si n.c. for 48 h, then the expressions of DNMT3A mRNA and protein were measured. **D** The CVB3-infected cells were co-transfected with si-DNMT3A or si n.c. and miR-29b-3p inhibitor. A total of 48 h later, pyroptosis level of cardiomyocytes was evaluated by western blotting. *N* = 3, *p < 0.05, **p < 0.01, ***p < 0.001, and ****p < 0.0001

To confirm that DNMT3A mediates miR-29b-3p`s regulation of pyroptosis, we modulated the DNMT3A expression in CVB3-infected cells transfected with the miR-29b-3p inhibitor, followed by the assessment of pyroptosis associated proteins (NLRP3, ASC, cleaved caspase-1, GSDMD-N, and IL-1β). We found that the miR-29b-3p inhibitor markedly suppressed pyroptosis related proteins expression induced by CVB3, whereas the pyroptosis inhibitory effect was further enhanced when DNMT3A was overexpressed (Fig. 7A). Additionally, we synthesized small interfering RNA (si-DNMT3A) to downregulate the DNMT3A expression. qRT–PCR and western blot results confirmed that si-DNMT3A effectively diminished the expression of DNMT3A (Fig. 7B, C). Furthermore, when DNMT3A expression was downregulated by siRNA, the pyroptosis inhibitory impact of miR-29b-3p inhibitor were attenuated and reversed (Fig. 7D). These findings provide evidence that miR-29b-3p regulates pyroptosis via DNMT3A in CVB3 infected cardiomyocytes.

## Discussion

Our study proved the beneficial effects of knockdown miR-29b-3p expression against CVB3 induced VMC in mice, which could be explained partly by its property of antipyroptosis. Moreover, it was noted that the miR-29b-3p inhibitor suppressed cardiomyocyte pyroptosis through upregulating the expression of DNMT3A. These results suggest that targeting miR-29b-3p may offer an effective therapeutic approach for VMC.

Increasing studies have highlighted the vital function of miRNAs as gene expression switches in key processes of VMC [28, 37, 38]. For instance, miR-15 has been found to upregulate the levels of NLRP3 and caspase-1 p20, thereby promoting the activation of NLRP3 inflammasome and aggravating the inflammatory response of viral myocarditis [28]. In humans and susceptible mice, miR-21-5p is reported to be continuously and markedly elevated during acute myocarditis, with a notable correlation to left ventricular systolic dysfunction [39]. Additionally, the expression of miR-155 and miR-148a are found to be significantly increased in viral myocarditis, indicating their involvement in the immune response triggered by CVB3 [40]. In this study, through transcriptome sequencing analysis, we found many miRNAs in CVB3-infected heart tissue were significantly changed in comparison to the control group. Among these changes, the upregulation of miR-29b-3p was particularly notable and subsequently validated through qRT–PCR analysis.

A number of investigations have identified that miR-29b-3p express abnormally in various disease models, particularly those related to inflammation. It can target SPPY1, activate MAPK signaling pathway, and increase inflammation and oxidative stress in atherosclerotic models [41]. In the model of intestinal ischemia/reperfusion injury, miR-29b-3p is proved to inhibit inflammatory response through suppressing the expression of TNF receptor-associated factor 3 (TRAF3) [42]. However, its relationship with VMC has not been documented before. Our study revealed that miR-29b-3p antagomir alleviated heart injury post CVB3 infection, as evidenced by the reduced inflammatory infiltration and pyroptosis in the heart tissues, as well as increased cardiac function and survival rate in VMC mice. Nevertheless, the underlying molecular mechanism remains unclear.

It is widely known that inflammation played a key factor in the process of VMC [1, 43–45]. Pyroptosis, as a kind of inflammatory cell death, plays a crucial role in defending against viral infections [46–48]. The process of inflammatory caspase-mediated pyroptosis is characterized by the activation of NLRP3 inflammasome and the secretion of proinflammatory cytokines, which are crucial for initiating, amplifying, and sustaining inflammation [47, 49]. More and more evidence indicate NLRP3 inflammasome activation is associated with the development of various inflammatory diseases including viral myocarditis [17, 50–52]. In this study, we observed a decreased cell viability in CVB3 infected cardiomyocytes and the high expression of NLRP3 inflammasome in VMC mice, along with increased levels of proinflammatory cytokines IL-18 and IL-1β. In both in vitro and in vivo models of CVB3-induced VMC, we discovered that suppressing miR-29b-3p expression obviously weakened the upregulation and activation of NLRP3 inflammasomes (including NLRP3, ASC, and caspase-1), as demonstrated by the decreasing level of inflammatory mediator IL-1β. We also investigated the protein GSDMD, which is known to be crucial in pyroptosis pathway. It can be cleaved into GSDMD-N by proinflammatory protease caspase-1, which recruited and activated by NLRP3 inflammasome. Interestingly, our findings revealed that the suppression of miR-29b-3p effectively decreased the activation and cleavage of GSDMD caused by CVB3 infection. These results indicate that by inhibiting cardiomyocyte pyroptosis, the inhibition of miR-29b-3p could potentially enhance the management of VMC.

DNMT3A, a kind of DNA methyltransferase, plays a pivotal role in chromatin remodeling and gene expression regulation. The regulatory role of DNMT3A in viral infection has been discovered through multiple studies. DNMT3A’s binding to viral capsid protein VP26 during herpes simplex virus-1 (HSV-1) infection affects viral replication [53]. Additionally, overexpression of DNMT3A has been shown to suppress hepatitis B virus (HBV) transcription [54]. Furthermore, DNMT3A is essential in generating type I interferon and providing resistance against viral infection in mice, highlighting its significant role in antiviral immunity [55]. Genes that encode products involved in innate immunity and are regulated by DNMT3A and DNA methylation have the potential to be targeted for drug discovery in diseases related to infection.

In our research, bioinformatic analysis was carried out using multiple online miRNA target prediction databases to identified DNMT3A as a potential target of miR-29b-3p. To verify this, the gain/loss-of-function experiment was applied. Downregulating miR-29b-3p expression in mice by using the miR-29b-3p antagomir lead to an upregulation of DNMT3A expression. Likewise, manipulating miR-29b-3p levels with inhibitor or mimic in vitro could modulate intracellular DNMT3A expression levels. Additionally, other researchers also conducted a luciferase reporter assay and revealed the fact that miR-29b-3p could targets the 3′-UTRs of DNMT3A [56–58]. That means in our VMC model, DNMT3A is the target of miR-29b-3p.

Considering the possible role of DNMT3A played in viral infection and inflammation, we also detected the relationship between DNMT3A and cardiomyocytes pyroptosis. Our findings demonstrated that overexpression of DNMT3A reversed CVB3-induced pyroptosis and enhanced the myocardial protective prosperities of the miR-29b-3p inhibitor during CVB3 infection. Conversely, the pyroptosis inhibitory effect of miR-29b-3p inhibitor could be partially offset by si-DNMT3A. These results indicate that miR-29b-3p regulates pyroptosis in VMC by modulating its target gene, DNMT3A.

**Fig. 8:**
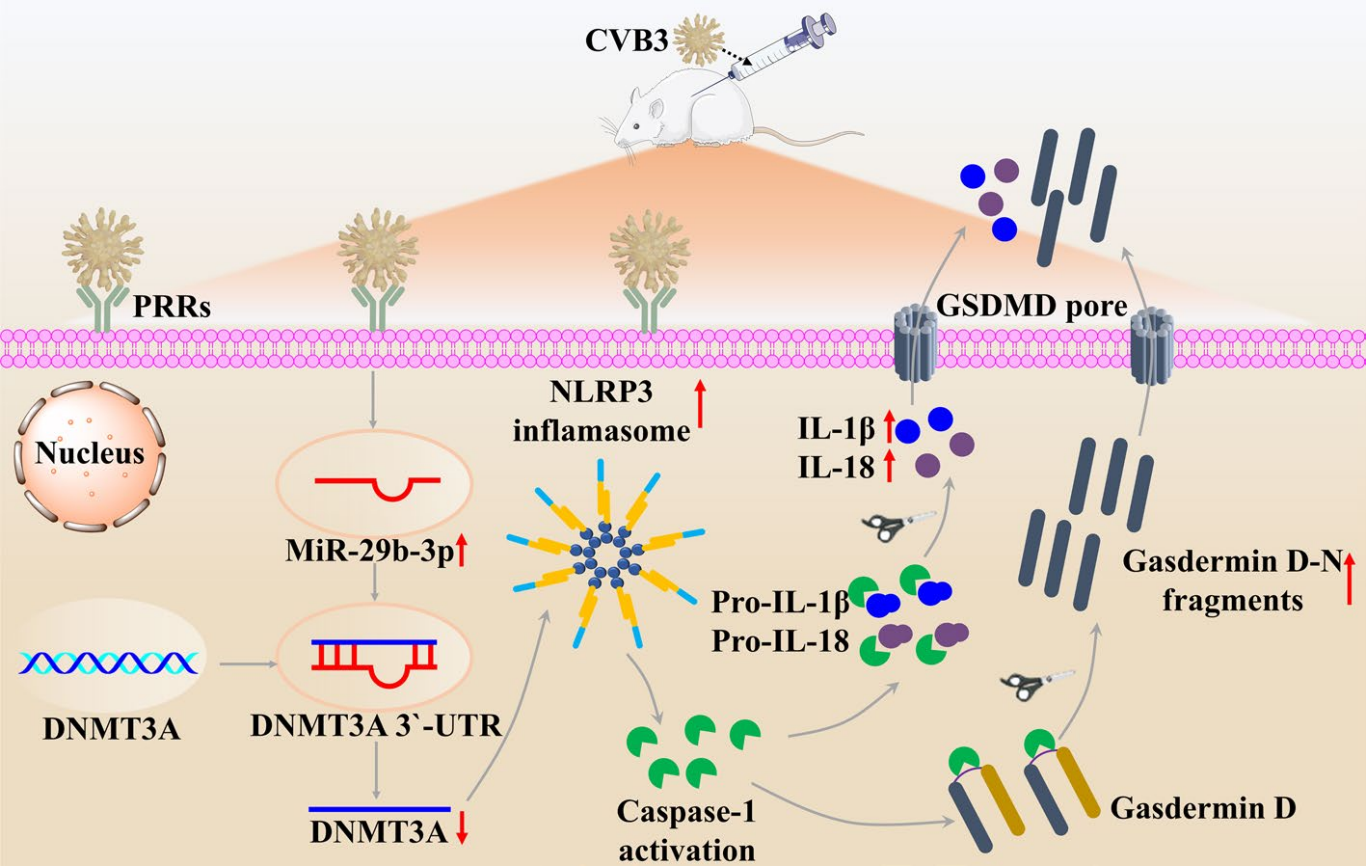
Schematic representation of CVB3-induced miR-29b-3p-mediated pyroptosis in cardiomyocytes. Infection with CVB3 leads to an upregulation in the expression of cellular miR-29b-3p, which subsequently downregulates the expression of DNMT3A by binding to the 3′-UTR of DNMT3A. This reduction in DNMT3A levels activates the NLRP3 inflammasome, resulting in the cleavage of pro-caspase-1 into its active form. The activated cleaved caspase-1 then cleaves GSDMD, generating N-terminal fragments that form pores in the cell membrane and trigger pyroptosis. Additionally, cleaved caspase-1 also activates pro-IL-1β and pro-IL-18, promoting their maturation and release. This process exacerbates the inflammatory damage to myocardial cells, leading to myocardial damage and a decline in cardiac function

To sum up, our research has demonstrated that the level of miR-29b-3p was dramatically upregulated in heart tissues of VMC mice. Furthermore, the downregulation of miR-29b-3p was found to alleviate VMC by suppressing pyroptosis via DNMT3A (Fig. 8). Our findings provide a previously unknown role of miR-29b-3p in the pathology of VMC and suggest its potential as a target for new therapeutic strategies against VMC.

## Conclusions

The present study demonstrates that miR-29b-3p could regulate pyroptosis in VMC by modulating its target gene, DNMT3A. It reveals a novel mechanism that CVB3 could induced cardiac injury through regulating miR-29b-3p/DNMT3A-mediated pyroptosis.

### Supplementary Information

**Additional file 1: Figure S1**. Part of cardiac function indicators in the VMC mice model. **A** The ratio of heart weight to body weight.** B**–**H **Other cardiac function indicators in the VMC model include heart rate, left ventricular end-diastolic anterior wall thickness (LVAW, d), left ventricular end-systolic anterior wall thickness (LVAW, s), left ventricular end-diastolic posterior wall thickness (LVPW, d), left ventricular end-systolic posterior wall thickness (LVPW, s), left ventricular end-diastolic internal diameter (LVID, d), and left ventricular end-systolic internal diameter (LVID, s). *N* = 3, *ns* significance is annotated as not significant, * *p * < 0.05, **p < 0.01, ***p < 0.001. **Figure S2. **The cell viability was detected by CCK8.** A** HL-1 cells were treated with different doses of CVB3 for 24 h, 48 h, and 72 h, respectively. ####*p* < 0.0001 represents the comparison with control at 48 h; ****p < 0.0001 represents the comparison with control at 72 h. **B **The morphological changes of cells post infection of CVB3 for 48 h. The magnification was 40×, and the scale bar was 100 μm. **C** Cells were treated as indicated for 48 h and the cell viability was evaluated by CCK8. *N* = 3, ****p < 0.0001. **Figure S3.** Part of cardiac function indicators in the VMC mice model. **A** The ratio of heart weight to body weight.** B**–**H** Other cardiac function indicators include heart rate, left ventricular end-diastolic anterior wall thickness (LVAW, d), left ventricular end-systolic anterior wall thickness (LVAW, s), left ventricular end-diastolic posterior wall thickness (LVPW, d), left ventricular end-systolic posterior wall thickness (LVPW, s), left ventricular end-diastolic internal diameter (LVID, d), and left ventricular end-systolic internal diameter (LVID, s). *N* = 3, #*p * < 0.05, ##*p* < 0.01, ###*p* < 0.001, ####*p* < 0.0001, *ns*, significance is annotated as not significant, *p < 0.05, **p  < 0.01, ****p < 0.0001

## Data Availability

No data were used for the research described in the article.

## Abbreviations

VMC: Viral myocarditis
DNMT3A: DNA methyltransferases 3A
CVB3: Coxsackievirus B3
CVDs: Cardiovascular diseases
PAMPs: Pathogen-associated molecular patterns
DAMPs: Danger-associated molecular patterns
IL-1β: Interleukin-1β
IL-18: Interleukin-18
GSDMD: Gasdermin D
GSDMD-N: Gasdermin D N-terminal fragment
miRNAs: MicroRNAs
PBS: Phosphate-buffered saline
SD rats: Sprague–Dawley rats
DMEM: Dulbecco’s modified Eagle medium
FBS: Fetal bovine serum
H&E: Hematoxylin and eosin
siRNA: Small interfering RNA
qRT–PCR: Quantitative real-time polymerase chain reaction
BSA: Bovine Serum Albumin
PCD: Programmed cell death
CO: Ventricular cardiac output
LVEF: Left ventricular ejection
LVFS: Left ventricular fraction shortening
SV: Stroke volume
TRAF3: TNF receptor-associated factor 3
HSV-1: Herpes simplex virus 1
HBV: Hepatitis B virus
