# Supplementary material for: miR-29b-3p regulates cardiomyocytes pyroptosis in CVB3-induced myocarditis through targeting DNMT3A
Source: Cell Mol Biol Lett. 2024 Apr 20;29:55. doi: 10.1186/s11658-024-00576-8 (PMC11031889; doi:10.1186/s11658-024-00576-8)
Supplement: Supplementary file 1 — Additional file 1: Figure S1. Part of cardiac function indicators in the VMC mice model. A The ratio of heart weight to body weight. B–H Other cardiac function indicators in the VMC model include heart rate, left ventricular end-diastolic anterior wall thickness (LVAW, d), left ventricular end-systolic anterior wall thickness (LVAW, s), left ventricular end-diastolic posterior wall thickness (LVPW, d), left ventricular end-systolic posterior wall thickness (LVPW, s), left ventricular end-diastolic internal diameter (LVID, d), and left ventricular end-systolic internal diameter (LVID, s). N = 3, ns significance is annotated as not significant, * p < 0.05, **p < 0.01, ***p < 0.001. Figure S2. The cell viability was detected by CCK8. A HL-1 cells were treated with different doses of CVB3 for 24 h, 48 h, and 72 h, respectively. ####p < 0.0001 represents the comparison with control at 48 h; ****p < 0.0001 represents the comparison with control at 72 h. B The morphological changes of cells post infection of CVB3 for 48 h. The magnification was 40×, and the scale bar was 100 μm. C Cells were treated as indicated for 48 h and the cell viability was evaluated by CCK8. N = 3, ****p < 0.0001. Figure S3. Part of cardiac function indicators in the VMC mice model. A The ratio of heart weight to body weight. B–H Other cardiac function indicators include heart rate, left ventricular end-diastolic anterior wall thickness (LVAW, d), left ventricular end-systolic anterior wall thickness (LVAW, s), left ventricular end-diastolic posterior wall thickness (LVPW, d), left ventricular end-systolic posterior wall thickness (LVPW, s), left ventricular end-diastolic internal diameter (LVID, d), and left ventricular end-systolic internal diameter (LVID, s). N = 3, #p < 0.05, ##p < 0.01, ###p < 0.001, ####p < 0.0001, ns, significance is annotated as not significant, *p < 0.05, **p < 0.01, ****p < 0.0001 [file 11658_2024_576_MOESM1_ESM.docx]

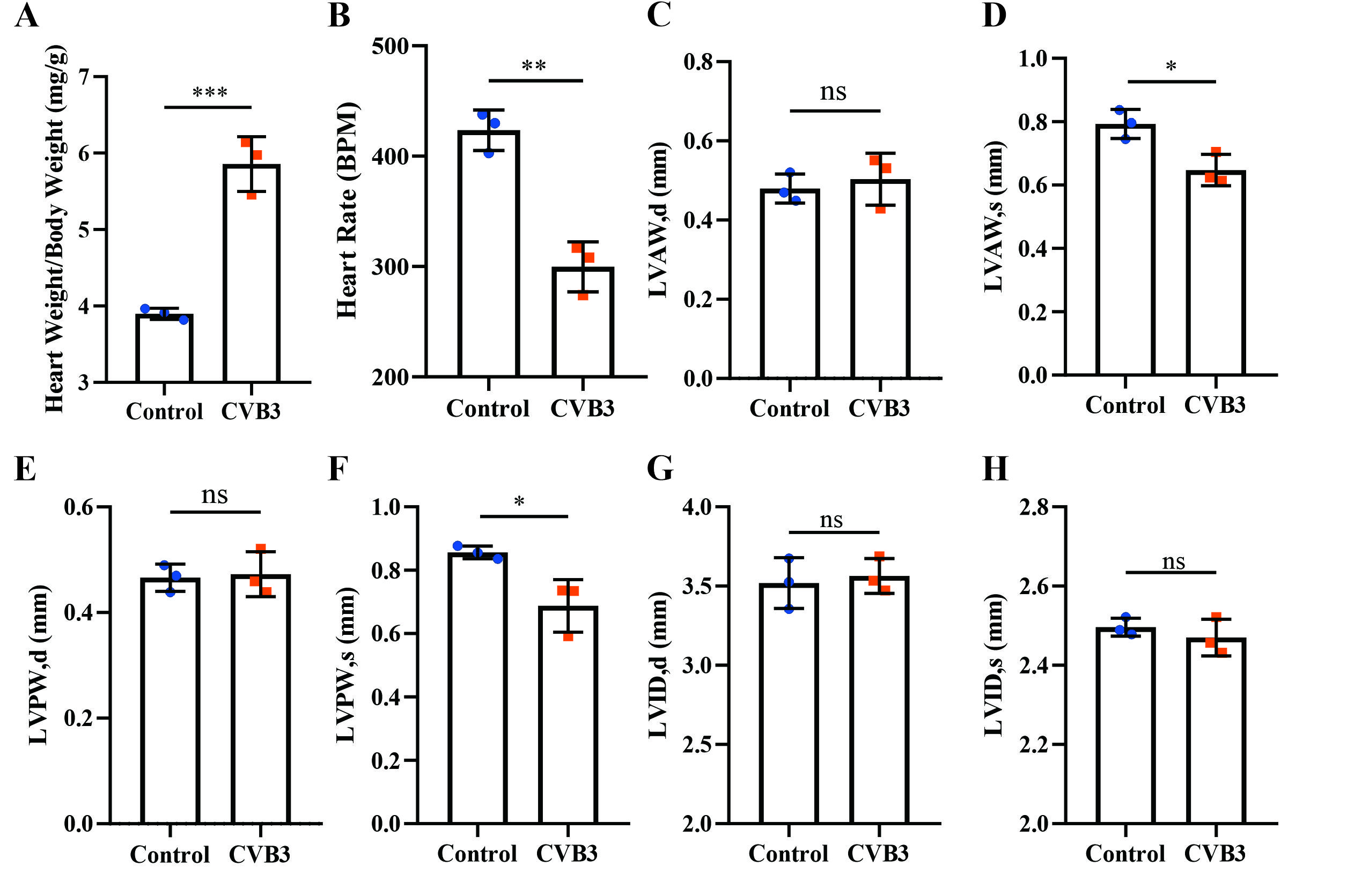


**Figure S1. Part of cardiac function indicators in the VMC mice model. (A)** The ratio of heart weight to body weight. **(B-H)** Other cardiac function indicators in the VMC model include heart rate, left ventricular end-diastolic anterior wall thickness (LVAW, d), left ventricular end-systolic anterior wall thickness (LVAW, s), left ventricular end-diastolic posterior wall thickness (LVPW, d), left ventricular end-systolic posterior wall thickness (LVPW, s), left ventricular end-diastolic internal diameter (LVID, d) and left ventricular end-systolic internal diameter (LVID, s). N=3, *ns*, significance is annotated as not significant, **p* ＜ 0.05*, **p* ＜ 0.01*, ***p* ＜ 0.001.


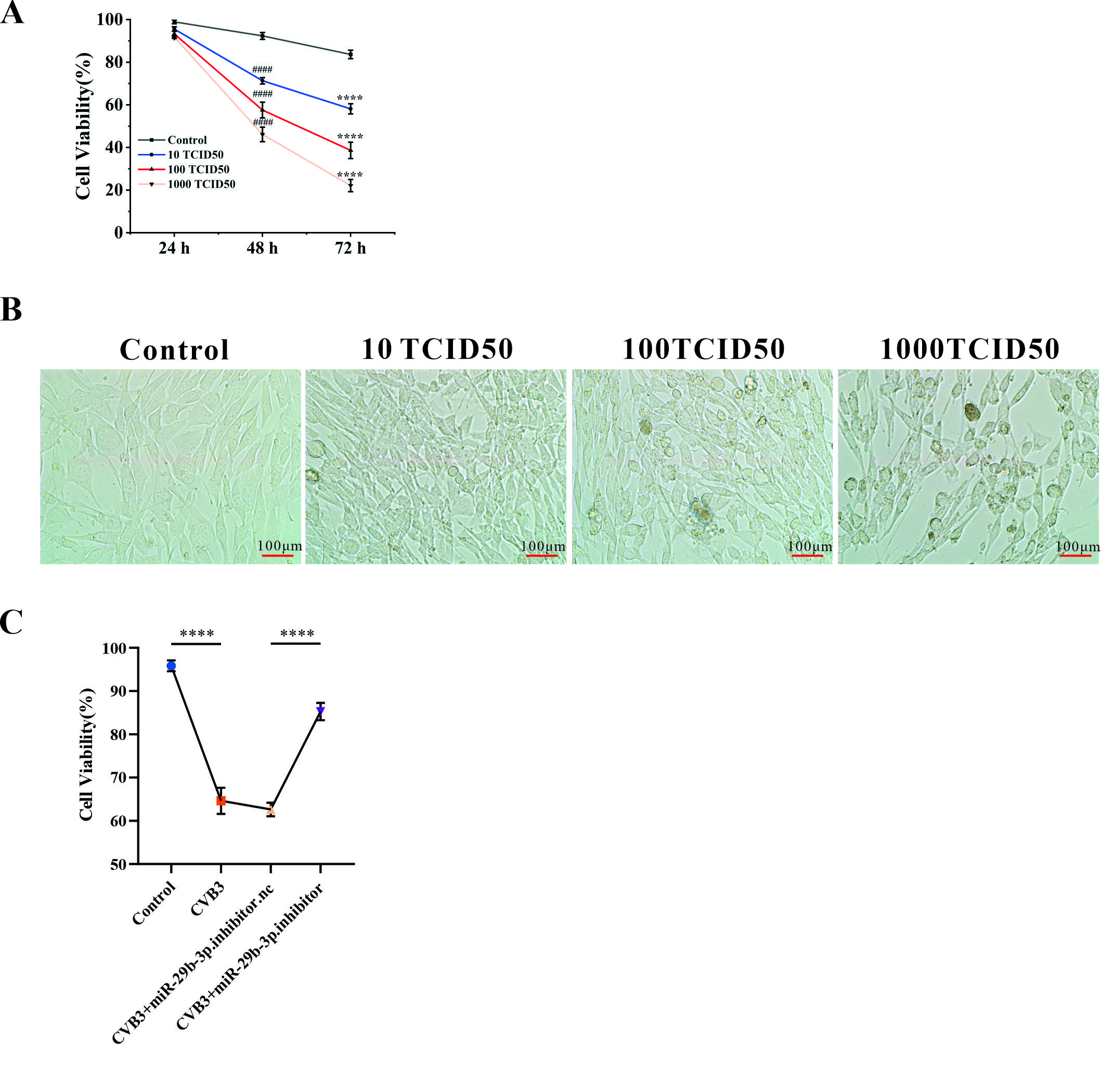


**Figure S2. The cell viability was detected by CCK8. (A)** HL-1 cells were treated with different doses of CVB3 for 24 h, 48 h, and 72 h, respectively. ####*p* ＜ 0.0001，represents the comparison with Control at 48h; *****p* ＜ 0.0001，represents the comparison with Control at 72h. **(B)** The morphological changes of cells post infection of CVB3 for 48 h. The magnification was 40 ×, and the scale bar was 100 μm. (**C**) Cells were treated as indicated for 48 h and the cell viability was evaluated by CCK8. N=3, *****p* ＜ 0.0001.


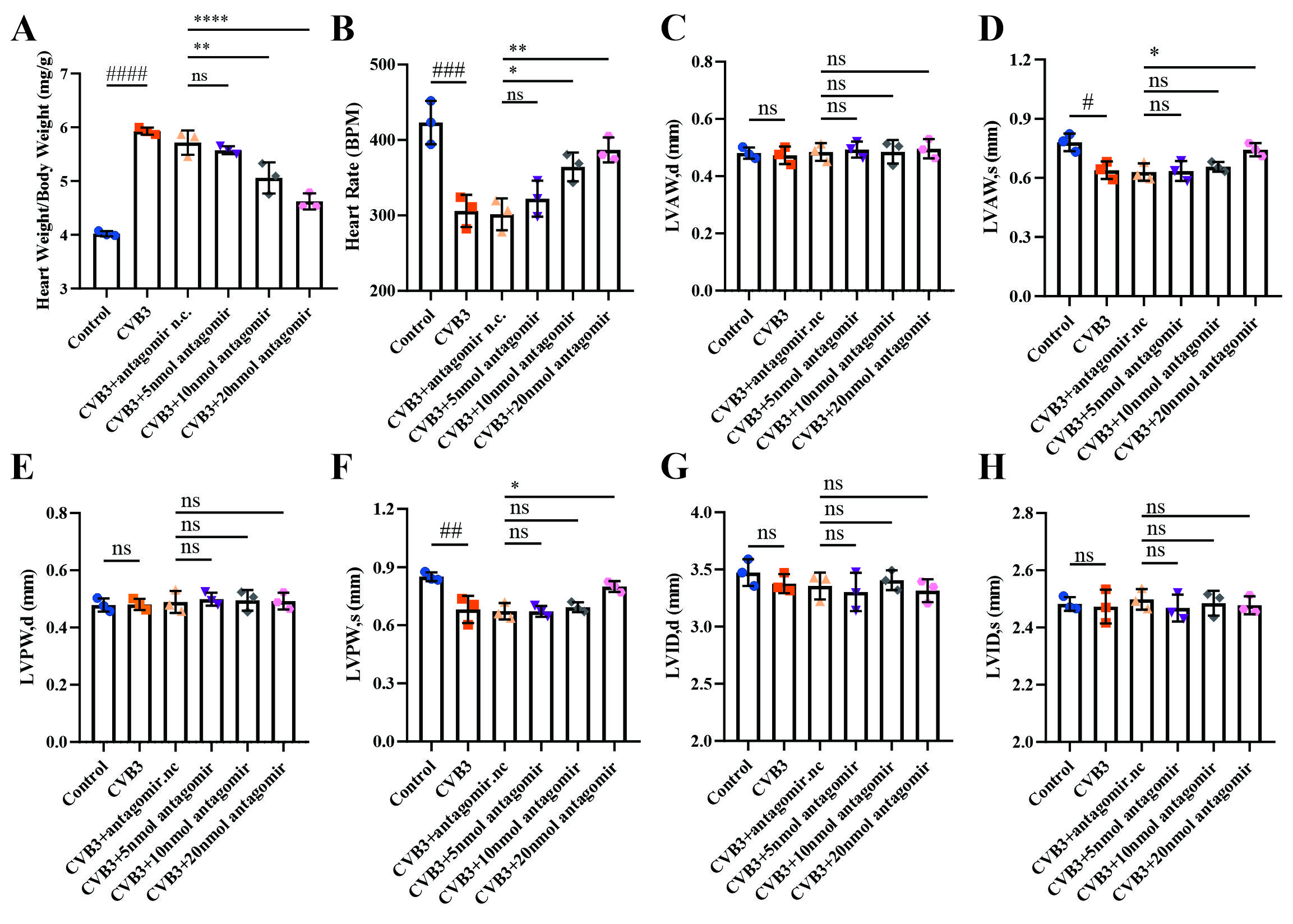


**Figure S3.** **Part of cardiac function indicators in the VMC mice model. (A)** The ratio of heart weight to body weight. **(B-H)** Other cardiac function indicators include heart rate, left ventricular end-diastolic anterior wall thickness (LVAW, d), left ventricular end-systolic anterior wall thickness (LVAW, s), left ventricular end-diastolic posterior wall thickness (LVPW, d), left ventricular end-systolic posterior wall thickness (LVPW, s), left ventricular end-diastolic internal diameter (LVID, d) and left ventricular end-systolic internal diameter (LVID, s). N=3, #*p* ＜ 0.05*,* ##*p* ＜ 0.01*,* ###*p* ＜ 0.001, ####*p* ＜ 0.0001, *ns*, significance is annotated as not significant, **p* ＜ 0.05*, **p* ＜ 0.01*, ****p* ＜ 0.0001.
